# Patient setup variation on Elekta Unity and its impact on adaptive planning

**DOI:** 10.1002/acm2.70016

**Published:** 2025-02-12

**Authors:** Maggie Yan, Erika Kollitz, Sheng‐Hsuan Sun, Kathryn Hitchcock, Alexandra De Leo, Amanda Schwarz, Luke Maloney, Jonathan Li, Chihray Liu, Guanghua Yan

**Affiliations:** ^1^ Department of Computer Information and Science Engineering University of Florida Gainesville Florida USA; ^2^ Department of Radiation Oncology University of Florida Gainesville Florida USA

**Keywords:** adaptive planning, Elekta Unity, MR‐linac, patient setup variation

## Abstract

**Purpose:**

The unique design of the MR‐linac may restrict the use of effective immobilization devices, resulting in significant patient setup variations (PSVs). The purpose of this study is to analyze the PSVs on the Elekta Unity system and investigate their impact on adaptive planning.

**Methods:**

The PSVs for 10 brain, 10 pancreas, five prostate, and five rectum patients previously treated on Elekta Unity were analyzed. The five prostate and five pancreas plans were selected to investigate the impact of PSVs on adaptive planning. The reference scans were shifted by 1, 2, and 3 cm in the left–right (LR) and superior–inferior (SI) directions to simulate PSVs. Both the adaptive‐to‐position (ATP) and adaptive‐to‐shape (ATS) workflows were executed. The adaptive planning time, number of monitor units (MUs), and dosimetric metrics quantifying target coverage and organ‐at‐risks (OARs) sparing were compared.

**Results:**

For brain treatments, the average/maximum PSVs were −0.2 ± 0.3 cm/0.8 cm (LR), 0.3 ± 0.7 cm/1.8 cm (SI), and 0.8 ± 0.7 cm/1.8 cm in the anterior–posterior (AP) direction. For pancreas treatments, the PSVs are −0.1 ± 1.0 cm/3.8 cm (LR), −0.1 ± 0.8 cm/3.5 cm (SI), and 0.3 ± 0.3 cm/1.3 cm (AP). Pelvis treatments had similar PSVs as pancreas treatments. The ATS workflow took two to three times longer than the ATP workflow. The only trend observed was that the plan MUs increased slightly (< 10%) with PSVs in the ATP workflow for prostate patients. Both workflows effectively reproduced target coverage and OAR sparing, regardless of the magnitude of the PSVs.

**Conclusions:**

Significant PSVs were observed on Elekta Unity due to suboptimal patient immobilization. Using prostate and pancreas treatments as examples, we demonstrated that adaptive planning can effectively accommodate such PSVs. Nevertheless, efforts should be made to minimize PSVs—particularly rotations—to mitigate intra‐fraction motion and reduce treatment time.

## 1 INTRODUCTION

In modern radiotherapy, patient immobilization is crucial to reduce daily patient setup variation (PSV) and improve treatment accuracy. Proper immobilization helps the patient effectively reproduce their position from simulation to treatment and maintain the position throughout the entire treatment course.^1^ The development of immobilization devices in radiotherapy has significantly evolved, driven by the need for increased treatment precision, patient comfort, and effective treatment delivery. For example, custom‐fit thermoplastic masks have been widely used for brain and head‐and‐neck radiotherapy, and sophisticated vacuum cushions and thermoplastic body molds can be made to conform to the patient's body contour to provide stable immobilization. Additionally, there is increasing interest in using indexed immobilization devices, which allow for precise calculation of treatment table parameters before treatment. This approach helps prevent treatment of the incorrect sites and streamlines the entire treatment workflow.^2^

In magnetic resonance‐guided radiation therapy (MRgRT), the integration of MR imaging with a linear accelerator (MR‐linac) presents unique challenges to patient immobilization.^3^ Due to the MR imaging components, the MR‐linac has a smaller gantry size than a conventional linac, placing limits on the lateral dimensions of immobilization devices. The need to include MR coils further restricts the available space for immobilization devices.^4^, ^5^ For example, the Elekta Unity system (Elekta AB, Stockholm, Sweden) has a bore diameter of only 70 cm. The anterior MR coil is integrated with the treatment couch, which makes it difficult to use custom‐fit vacuum cushions or thermoplastic body molds. The requirement of being MR‐compatible may also limit the use of some immobilization devices. On the Unity system at our institution, customized thermoplastic masks are still used for brain and head‐and‐neck (HN) patients with either MR‐compatible brain or HN boards, respectively, which provides similar immobilization as is available on conventional linacs. However, for thorax, abdominal, and pelvic treatments, only a plain pad is placed underneath the patient with a knee roll and a foot board to provide cushion and stability. The immobilization for these treatments can be compromised due to the constraints imposed by the MR‐linac.

These compromises on patient immobilization make it harder for the patient to reproduce and hold their desired treatment position. The absence of lateral lasers on the Unity system and its inability to translate or rotate the treatment couch make it difficult to accurately and consistently position the patient. As a result, significant daily PSV can be expected for these treatments. Unlike conventional linacs, the Elekta Unity does not permit movement of the treatment couch to adjust for PSVs. Instead, it provides the option of treatment plan adaptation to address these issues.^6^ The Elekta Unity system offers two types of adaptive workflows: adapt to position (ATP) and adapt to shape (ATS).^6^ The ATP workflow is used when there is only negligible change in the size or shape of the treatment target or nearby organs‐at‐risk (OARs) with respect to the references. In these cases, PSVs are due primarily to setup uncertainties and only occur in the translational directions. The online optimizer accounts for these variations by adapting the multileaf collimator (MLC) leaf positions and reoptimizing the weights of individual segments in each beam. The ATS workflow can be used when there is significant change in the size and shape of the treatment target or nearby OARs. The contours of these structures can be edited by the clinicians in real‐time, followed by a ``warm start'' optimization based on the reference plan. The ATS workflow is more time‐consuming than the ATP workflow. Both adaptive workflows last significantly longer than the non‐adaptive workflow on a conventional linac. Reducing the time in the adaptive workflow is crucial, particularly because patients are confined in tight spaces with suboptimal immobilization. Any delay increases the risk of intrafraction motion, which can compromise treatment accuracy.^7^, ^8^ Additionally, it is clinically important to determine whether the magnitude of PSVs impacts the effectiveness of adaptive planning in terms of dosimetry, specifically, whether adapted plans maintain target coverage while preserving OAR sparing.

This study has two primary aims. First, we analyze PSVs on the Elekta Unity system across various treatment sites, including brain, abdomen, and pelvis. By examining these variations, we aim to understand how different factors contribute to setup discrepancies and their effects on treatment delivery. Second, we investigate the impact of the magnitude of the PSVs on adaptive planning. Elekta Unity relies on adaptive planning to compensate for the PSVs. Therefore, understanding such impact is crucial for identifying potential areas for improvement. Insights gained from this analysis will help determine the necessary adjustments to improve the workflow while maintaining treatment accuracy. By addressing these issues, we can work towards optimizing the system's performance, ultimately ensuring that patient treatments are delivered with the highest precision and efficacy.

## 2 METHODS AND MATERIALS

### 2.1 Immobilization on Elekta Unity

The Elekta Unity integrates a 1.5 T MR scanner with a 7 MV flattening‐filter‐free linac, providing high soft tissue contrast MR imaging and the possibility of online plan adaptation for each treatment fraction.^9^ Currently, it only supports the use of intensity‐modulated radiotherapy (IMRT). It has a source‐to‐axis distance of 143.5 cm with 80 leaf pairs (0.72 cm width at isocenter) providing a maximum field size of 57.4 × 22.0 cm^2^. The collimator is not allowed to rotate. The treatment couch only moves in the longitudinal direction for moving the patient in and out of the bore. Once patient setup is completed and daily imaging starts, the couch cannot be moved to compensate for setup variations. In our institution, the system has been used to treat various disease sites including brain, head & neck, esophagus, abdomen, pelvis, and so forth.

For brain treatments, thermoplastic masks are used in conjunction with a brain board, which is indexed to the treatment couch. Similarly, HN treatments use thermoplastic masks with an indexed HN board, equipped with straps to depress the patient's shoulders. Both the brain and HN boards are provided by QFix (Avondale, PA, USA). For both types of treatments, the anterior MR receiver coil is adjusted to the lowest feasible position to ensure the best image quality.

Thoracic, abdominal, and pelvic treatments on the Elekta Unity share similar setups. The patient is positioned with a head holder of appropriate size, which is indexed to the treatment couch. A 4 cm thick cushion pad is placed beneath the patient for added comfort. A knee roll and a foot board (CIVCO Medical Solutions, Orange City, IA, USA) are used for stability. Since the head holder is indexed to the couch, the foot board is not indexed to allow flexibility in positioning. Depending on the treatment site, the patient's hands are either by their sides with arm support, or on their chest holding a ring.

In CT/MR simulation, three MR‐compatible BBs are placed on the mask for brain/HN and on the patient's skin for other anatomical sites with the aid of in‐room laser guidance. In the Monaco treatment planning system (Elekta AB, Stockholm, Sweden), the BBs are identified on the scans to calculate the required longitudinal couch parameters. During patient setup for treatment on Elekta Unity, the anterior BB is aligned with the sagittal laser to center the patient laterally, while the two lateral BBs are aligned with the couch index to position the patient in the longitudinal direction.

### 2.2 PSV

In each treatment fraction, a localization MR image is obtained, which is rigidly registered with the reference image scan acquired during simulation. A clipbox representing the volume of interest centering around the treatment target is defined to guide the local rigid registration. Since there is no mechanism to correct rotational setup variations, rigid image registration is used and only translational shifts are calculated. These shifts, which represent the PSVs, are recorded for every treatment fraction of every patient in our institution.

The PSVs of 10 brain patients, 10 pancreas patients, and 10 pelvis patients (five prostate and five rectum) previously treated in our institution were included in this analysis. In the brain patient cohort, three received 25 Gy in five fractions, four received 60 Gy in 30 fractions, and three received 45 Gy in 25 fractions, totaling 210 fractions. For the pancreas patients, four received 40 Gy in five fractions and the remaining six received 50.4 Gy in 28 fractions, totaling 168 fractions. The five prostate patients received 60 Gy in 20 fractions and the five rectum patients received 25 Gy in five fractions, totaling 125 fractions for pelvis treatments.

### 2.3 Impact on adaptive planning

The treatment plans of the five prostate patients and five pancreas patients were used to study the impact of the magnitude of the PSVs on adaptive planning. Each of the plans used either 11 or 13 IMRT beams with a maximum of 80 total segments.

To simulate various PSVs, the reference MR scan acquired in simulation was altered with known artificial shifts and re‐imported into Monaco to imitate a daily scan. The two scans were first rigidly registered to determine the isocenter shift, then both ATP and ATS workflows were executed in the offline mode. In the ATP workflow, adaptive planning was performed on the reference scan. The shape and weight of each MLC segment were reoptimized to account for the shifts. Five optimization iterations were completed without interruption. In the ATS workflow, the reference contours were first propagated to the daily scan using deformable registration function. At this point in the clinical workflow, the contours would be edited by the physicians to account for anatomical changes. Edits were omitted here since the two image sets were identical and it did not contribute additional value to our study findings. Monaco provides two options to start ATS optimization: from segments or from fluence. When the optimization starts from segments, it inherits the segments from the reference plan; otherwise, the optimizer first finds the optimal fluence to achieve the predefined IMRT objectives, then performs leaf sequencing to produce a deliverable plan. We used the fluence option because it can produce superior plans, particularly in cases involving contour changes.

Seven different intentional shift combinations were introduced: S0(0,0,0), S1(1,1,0.5), S2(2,2,0.5), S3(3,3,0.5), S4(1,0,0.5), S5(2,0,0.5), and S6(3,0,0.5). The numbers, in centimeters, denote the shifts in the left–right (LR), superior–inferior (SI), and anterior–posterior (AP) directions, respectively. S0 had zero translational shift in each direction, representing the baseline. The others had 0.5 cm AP shift because our data showed that there appeared to be a systematic AP setup variation with an average of 0.5 cm. The maximum shift in the LR and SI direction observed in our analysis was around 3 cm. Therefore, we studied the impact when there were 1, 2, and 3 cm shifts in both LR and SI directions. The last three combinations had zero shift in the SI direction to investigate whether there were any advantages in the adaptive planning if the couch could move in the SI direction to correct setup variations.

Unlike the ATP workflow, the ATS workflow allows users to adjust optimization constraints during adaptive planning. However, no adjustments were made in this study to ensure a fair comparison between the two workflows. In both workflows, the reoptimized plans were rescaled to cover 95% of the planning target volume (PTV) with the prescribed dose (60 Gy for prostate plans and 50.4 Gy for pancreas plans). The dose scaling was consistently applied to both the reference plans and the adaptive plans with a scaling factor which was typically under 3%. We recorded the warm‐start optimization time as reported by the treatment planning system optimization console and the total number of monitor units (MUs) for each adaptive plan. Dosimetrically, we recorded the maximum dose received by 0.5 cc (D_0.5cc_) of the PTV and the minimum dose received by 99% of the PTV (D_99%_), representing hotspots and target coverage, respectively. For prostate plans, we recorded D_0.5cc_ and relative volume of the rectum and bladder receiving 60, 50, 40, and 30 Gy. For pancreas, we recorded D_5cc_ of the stomach, relative volume of both small bowel and large bowel receiving 40 Gy, dose received by 50% of both kidneys (D_50%_) in addition to a few dose criteria on spinal cord, liver, and esophagus.

## 3 RESULTS

**FIGURE 1:**
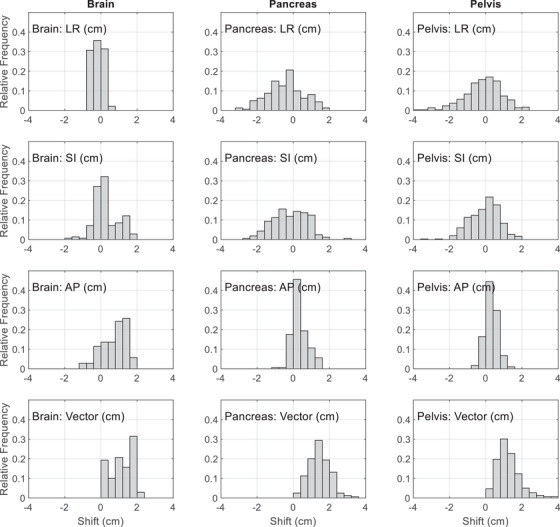
PSVs on Elekta Unity for brain, pancreas, and pelvis treatments in the LR, SI, and AP directions. The distribution of the 3D vectors of the variations are also shown in the last row. AP, anterior–posterior; LR, left–right; PSVs, patient setup variations; SI, superior–inferior.

Figure 1 shows the PSVs for patients treated on the Elekta Unity. For brain treatment, the average PSVs were −0.2 ± 0.3 cm, 0.3 ± 0.7 cm, and 0.8 ± 0.7 cm, while the maximum was 0.8, 1.8, and 1.8 cm in the LR, SI, and AP directions, respectively. The three‐dimensional (3D) vector had a mean magnitude of 1.2 ± 0.6 cm and a maximum of 2.1 cm. Pancreas treatments shared similar PSVs with pelvis treatments. The mean/maximum PSVs were −0.1 ± 1.0 cm/3.8 cm for pancreas and −0.4 ± 1.0 cm/3.1 cm for pelvis in the LR direction, −0.1 ± 0.8 cm/3.5 cm for pancreas and −0.2 ± 1.0 cm/3.0 cm for pelvis in the SI direction, and 0.3 ± 0.3 cm/1.3 cm for pancreas and 0.3 ± 0.4 cm/1.4 cm for pelvis in the AP direction. The 3D vector had a mean magnitude of 1.2 ± 0.6 cm and a maximum of 3.9 cm for pancreas, and a mean of 1.5 ± 0.6 cm and a maximum of 3.3 cm for pelvis. For pancreas and pelvis treatments, the overall variations in the AP direction were smaller than those in the LR/SI directions. Brian treatments had smaller overall variations than both pancreas and pelvis treatments in the LR/SI directions.

**FIGURE 2:**
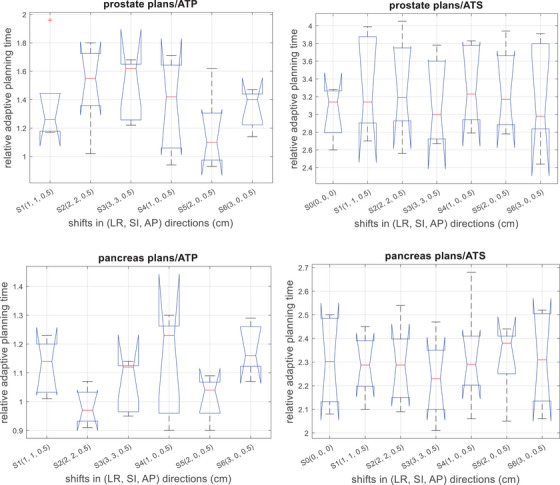
Relative adaptive planning time for adaptive planning in the ATP/ATS workflow as a function of setup variations for prostate and pancreas patients. The times were normalized with respect to the time needed in the ATP workflow when there were no shifts. ATP, adaptive‐to‐position; ATS, adaptive‐to‐shape.

Figure 2 shows the impact of the PSVs on adaptive planning time for both prostate and pancreas patients. For the prostate patients, in the ATP workflow, the mean relative adaptive planning time was 1.37 ± 0.33, 1.51 ± 0.30, 1.49 ± 0.22, 1.36 ± 0.33, 1.17 ± 0.27, and 1.34 ± 0.14 for shift S1 through S6, respectively; in the ATS workflow, these numbers were 3.33 ± 0.56, 3.30 ± 0.57, 3.25 ± 0.49, 3.32 ± 0.46, 3.28 ± 0.48, and 3.21 ± 0.61. The *p*‐values of the one‐way analysis of variance (ANOVA) on the adaptive planning time across the tested shift magnitudes were 0.45 for the ATP workflow and 0.99 for the ATS workflow. The ratio of the mean relative adaptive planning time between the ATS and the ATP workflow for the five prostate patients was 2.83 (263.8 vs. 93.1 s), 2.29 (242.3 vs. 105.9 s), 2.56 (264.3 vs. 103.4 s), 2.18 (264.0 vs. 120.9 s), and 2.32 (254.3 vs. 109.6 s), respectively. For the pancreas patients, in the ATP workflow, the mean relative adaptive planning time was 1.12 ± 0.09, 0.98 ± 0.06, 1.06 ± 0.09, 1.13 ± 0.18, 1.01 ± 0.08, and 1.18 ± 0.09 for shift S1 through S6, respectively; in the ATS workflow, these numbers were 2.29 ± 0.13, 2.29 ± 0.17, 2.23 ± 0.17, 2.32 ± 0.23, 2.32 ± 0.16, and 2.31 ± 0.20. The ANOVA test across the studied shifts yielded *p* = 0.06 for the ATP workflow and *p* = 0.97 for the ATS workflow. The ratio of the mean relative adaptive planning time between the ATS and the ATP workflow for the five pancreas patients was 2.12 (318.3 vs. 150.0 s), 1.84 (294.6 vs. 159.8 s), 2.19 (295.3 vs. 135.1 s), 2.35 (365.0 vs. 155.3 s), and 2.03 (293.8 vs. 144.9 s), respectively.

**FIGURE 3:**
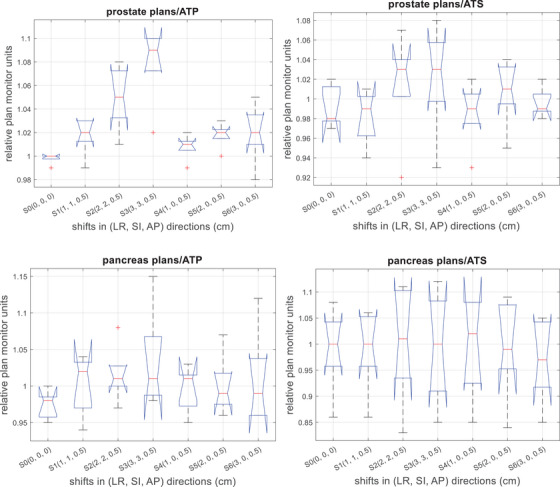
Relative MUs for adaptive planning in the ATP/ATS workflow as a function of setup variations for prostate and pancreas patients. The MUs were normalized with respect to the MU in the respective reference plan. ATP, adaptive‐to‐position; ATS, adaptive‐to‐shape; MU, monitor units.

Figure 3 shows the impact of the PSVs on the MUs of the adaptive plans in both workflows. The MUs were normalized to the MU in the reference plan. For the prostate plans, in the ATP workflow, the averages of the relative MUs were 1.02 ± 0.02, 1.05 ± 0.03, 1.08 ± 0.03, 1.01 ± 0.01, 1.02 ± 0.01, and 1.02 ± 0.03 for shift S1 through S6, respectively; in the ATS workflow, these numbers were 0.98 ± 0.03, 1.02 ± 0.06, 1.01 ± 0.06, 0.99 ± 0.03, 1.01 ± 0.03, and 1.00 ± 0.02. The *p*‐values of the ANOVA test were 0.02 and 0.55 for the ATP and ATS workflow, respectively. For the pancreas plans, in the ATP workflow, the averages of the relative MUs were 1.00 ± 0.04, 1.02 ± 0.04, 1.03 ± 0.07, 1.00 ± 0.03, 1.00 ± 0.04, and 1.01 ± 0.07 for shift S1 through S6, respectively; these numbers were 0.99 ± 0.08, 1.00 ± 0.11, 0.99 ± 0.11, 1.00 ± 0.10, 1.00 ± 0.10, and 0.97 ± 0.08 for the ATS workflow. The ANOVA test yielded *p*‐values of 0.85 for the ATP workflow and 0.99 for the ATS workflow.

**FIGURE 4:**
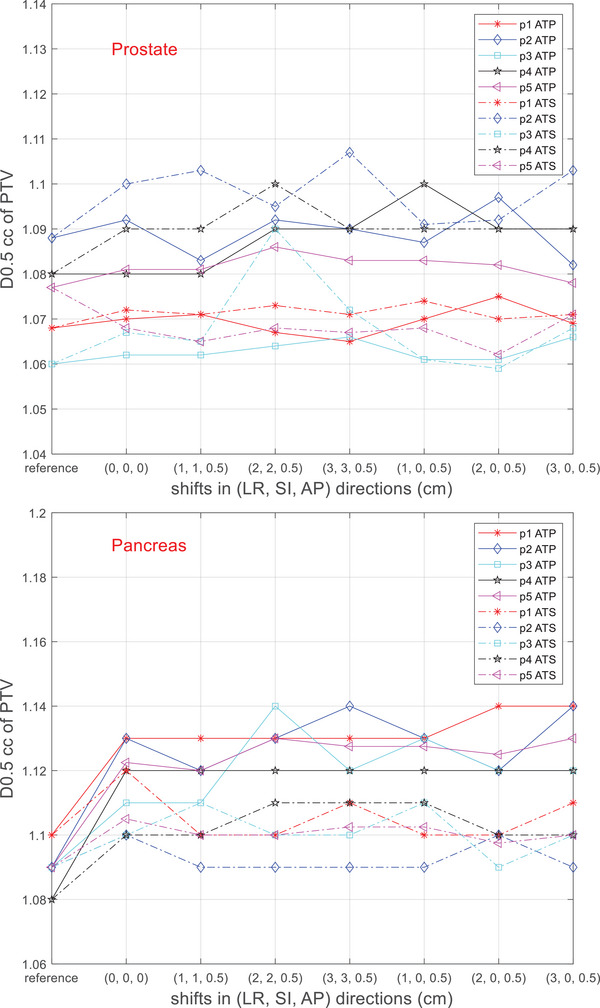
Maximum relative dose to 0.5 cc of the PTV (D0.5cc) in the adaptive plans. All the values were normalized to the prescription dose. The values at “reference” indicate the D0.5cc values in the respective reference plans. PTV, planning target volume.

**FIGURE 5:**
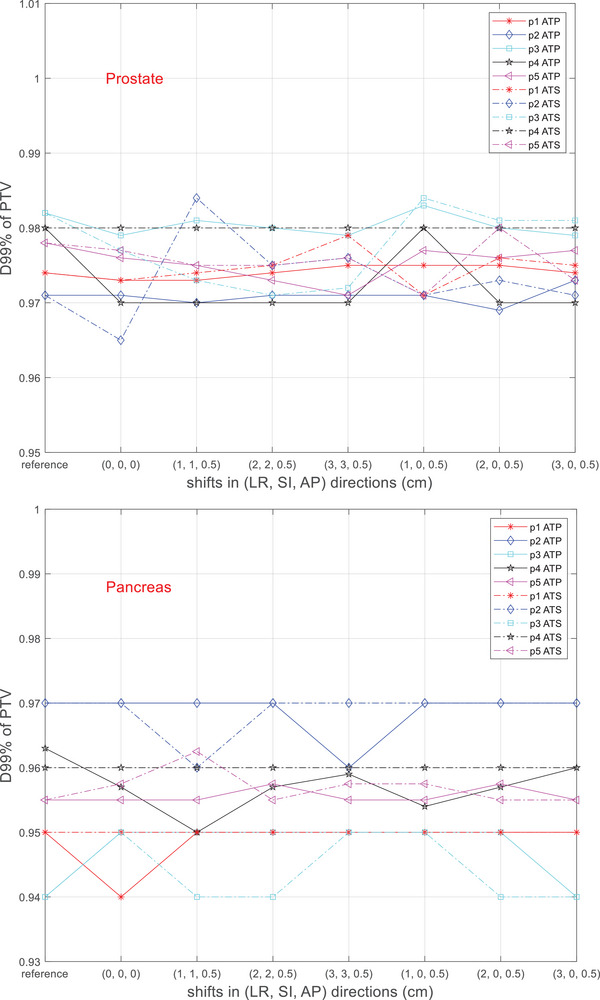
Minimum dose to 99% of the PTV (D_99%_) in the adaptive plans. All the values were normalized to the prescription dose. The values at “reference” indicate the D_99%_ values in the respective reference plans. PTV, planning target volume.

Figures 4 and 5 show the D_0.5cc_ and D_99%_ of the PTV in the adaptive plans as the magnitude of shifts changes. In both the ATP and the ATS workflows, D_0.5cc_ and D_99%_ of all the patients varied by no more than 3% and 2%, respectively. Both adaptive workflows maintained similar PTV coverage as the reference plan, regardless of the magnitude of the PSVs.

**FIGURE 6:**
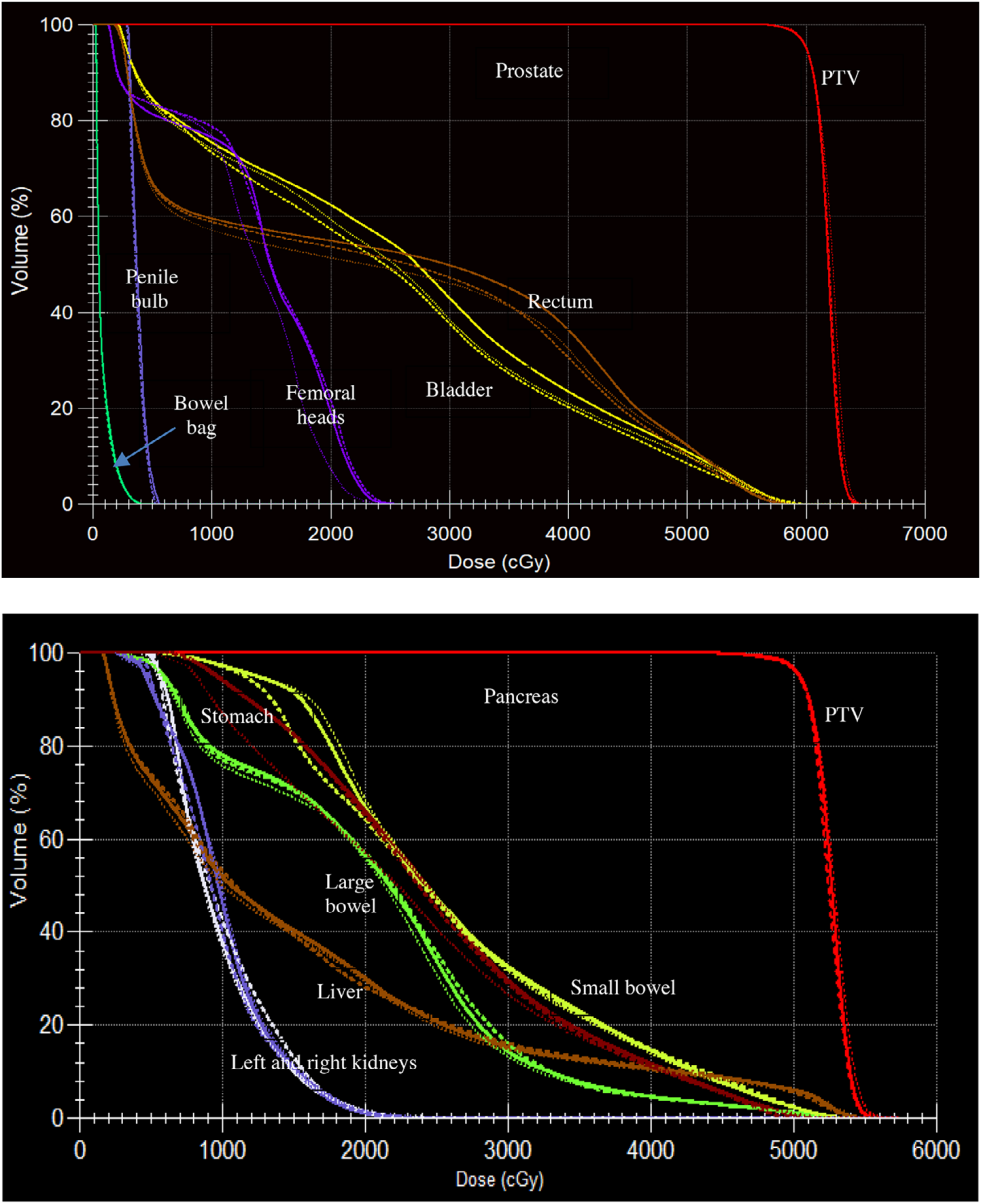
Dose volume histogram of the reference plan (solid lines), an ATP plan (dashed lines), and an ATS plan (dotted lines) for a prostate and a pancreas patient. The intentional shifts were (3, 3, 0.5) cm for the adaptive plans. ATP, adaptive‐to‐position, ATS, adaptive‐to‐shape.

For the prostate patients, the OAR sparing was slightly improved in the majority of the cases. The D_0.5cc_ of both the rectum and the bladder remained within 2% of the reference values. The V_60_ _Gy_ and V_50_ _Gy_ values decreased by 1%–2%, while V_40_ _Gy_ and V_30_ _Gy_ values were reduced by approximately 3%–5% in most cases. For the pancreas patients, all the adaptive plans nearly reproduced the OAR sparing achieved with the respective reference plans except small difference in the low dose region. For example, the D_5cc_ of the stomach was within 2% of the reference value in all the adaptive plans, but the ATS plan showed improvement in the dose area under 30 Gy. As examples, Figure 6 shows the dose volume histogram comparisons of reference plans and representative adaptive plans for a prostate patient and a pancreas patient. For the adaptive plans, the shift was (3, 3, 0.5) cm. No clear trend in OAR sparing was observed in relation to the magnitude of the shifts.

## 4 DISCUSSION

Significant PSVs were observed on the Elekta Unity, partially due to the compromise in patient immobilization. In a previous study from the same institution, the authors found that the inter‐fraction setup variations of 275 patients treated on conventional linacs were within 1.0 cm for 95% of the treatment fractions across all the treatment sites, evaluated with cone‐beam CT.^2^ On the Elekta Unity, without customized body molds and lateral lasers, it is challenging to set the patient straight, leading to larger PSVs in the LR directions for the pancreas/pelvis treatments. Other than suboptimal immobilization devices, there are a few unique factors that could have contributed to the PSVs. First, there were uncertainties in using the lateral BBs to establish the patient's position relative to the indexed couch. During simulation, the treatment couch was shifted longitudinally until a full index number aligned with the room laser, at which point the BBs were placed, and skin tattoos were applied. However, due to the softness of the skin, these tattoos could shift relative to the treatment target over time. This introduced uncertainty in patient positioning, as the couch's longitudinal coordinate was determined based on the indexed couch number. Additionally, the sagittal laser, used to align and center the patient laterally, lacks stability, which may contribute to setup uncertainties. Second, in the treatment planning for Elekta Unity, a set of contours representing the treatment couch were inserted to ensure accurate dose calculation. The vertical location of the couch contours was determined empirically: for pancreas and pelvis treatments, a 0.5 cm spacing between the patient's skin and the couch top was used to account for the thickness of the compressed thin pad; for brain treatments, we initially used a 4.5 cm spacing and switched to a 5.0 cm spacing later. This could explain why the distribution of deviations in the AP direction was skewed towards one side for the pancreas/pelvis treatments, while it was nearly bimodal for the brain treatments. Additionally, for pancreas/pelvis treatments, internal organ motion and variations in bladder and rectum filling can significantly impact the PSVs in all three directions.

To ensure the quality of the adapted plans and demonstrate maximum possible impact of the PSVs, we used the most complex methods available in Monaco for both adaptive workflows: optimize weights and shapes from segments for ATP and optimize weights and shapes from fluence for ATS. The ATP workflow shifts the segments from the reference plans prior to further optimization, while the ATS workflow discards the reference segments and starts a fluence optimization from scratch. This could have implications on the adaptative planning time, the number of MUs of the plans (which directly affects the delivery time), and the plan dosimetric quality. As expected, our results showed that the ATS workflow took significantly more time in adapting the plans than the ATP workflow. The adaptive planning time did not increase as the magnitude of the PSVs increased in either workflow. This is not surprising, as neither shifting the segments nor optimizing the fluence was affected by the extent of the isocenter shift. Regarding relative plan MUs, a statistically significant difference was observed exclusively in the ATP workflow for prostate patients, where a clear trend of increasing MUs was associated with greater setup variations. The maximum increase in the MUs (10%) occurred when there were maximum variations (3 cm in both the LR and SI directions). For the prostate plans, all the adapted plans met our target coverage goals (D_0.5cc _< 110% of the prescribed dose and D_99% _> 93% of the prescribed dose) except three ATS plans for patient 2, where the D_0.5cc_ value slightly exceeded the constraints. Winkel et al. did a similar study comparing the two adaptive workflows and found that the ATP workflow often resulted in plans lacking sufficient target coverage, while the ATS workflow was more robust.^6^ Our adaptive plans did not have coverage issues because all the plans were scaled based on the prescribed dose (D_95%_ = prescription). In more complex treatments like the pancreas plans, the dose scaling may cause the hotspots to exceed the constraints (refer to Figure 4). In our clinical workflow, relevant IMRT objectives are adjusted to suppress the PTV hotspots in the ATS workflow. All the adaptive plans were able to maintain or slightly improve OAR sparing, regardless of the magnitude of the PSVs.

The findings of this study suggest that although significant PSVs are observed on the Elekta Unity, adaptive planning can effectively account for them. However, every effort should still be made to ensure optimal patient immobilization for several reasons. First, the rigid registration between the daily scan and the reference scan calculates translational shifts but does not account for rotations. Without lateral lasers and a customized body mold, significant rotations can occur despite careful attention by the therapists. Rotation errors can also occur on the brain/HN patients if their thermoplastic masks no longer fit properly. In these scenarios, the patient either needs to be repositioned or the ATS workflow must be used, both of which result in extended treatment time. Second, this study focuses solely on inter‐fractional setup variations and does not account for intra‐fractional motion. While the recently released comprehensive motion management system can correct systematic shifts during treatment, it also extends the overall treatment time. Therefore, it is essential for the patient to maintain the same position throughout imaging, adaptive planning, and treatment delivery. Proper immobilization ensuring both comfort and stability for the patient is crucial. Third, setup variations exceeding 5 cm will halt the adaptive workflow, requiring the patient to be repositioned and rescanned, which results in treatment delays. Additionally, the Elekta Unity has a limited field size in the SI direction (22 cm). For treatment targets that just fit within the limit, setup variations of a few centimeters can lead to inaccuracy in dose calculation or even halt the adaptive workflow.

A limitation of this study is the small sample size of IMRT plans, with only five plans per site. This may reduce the study's statistical power to detect subtle differences. For instance, in the ANOVA test of relative adaptive planning time for the ATP workflow with the pancreas plans, the *p*‐value of 0.06 suggests a potential trend but falls short of statistical significance. Further investigation with a larger sample may be warranted to draw more definite conclusions.

For thoracic, abdominal, and pelvic treatments, the CIVCO knee roll and footboard are used in place of those provided by Elekta. The CIVCO devices are preferred due to their enhanced patient comfort, attributed to their softer material. Additionally, a 4 cm thick cushion pad is placed under the patient to further improve comfort. However, unlike the Elekta knee roll and footboard, the CIVCO devices cannot be indexed to the treatment couch, which may increase PSV in the SI and LR directions. To address this limitation, a head holder indexed to the treatment couch is employed to ensure consistent positioning in the SI direction. Reproducibility in the LR direction is achieved using the sagittal laser, which is checked daily against a reference mark on the treatment couch to maintain consistency.

This study focused on prostate and pancreas IMRT plans. The prostate plans were selected due to the relatively straightforward nature of the cases involved.^10^ Specifically, because there was no lymph node involvement, the PTV was relatively regular in shape and centrally positioned. The simplicity facilitated a more straightforward analysis of the setup variations and their impact on adaptive planning. On the other hand, the pancreas plans were relatively more complicated due to the presence of many nearby OARs, which led to longer adaptive planning time and slightly worse plan quality (hotspots in PTV > 10%). It is still important to note that these findings may not be directly applicable to more complex cases. For instance, treatments involving irregular PTV shapes at off‐center locations may present additional challenges that could influence the extent and impact of the PSVs in ways not captured by the current study. It is also worth noting that all the reference plans readily met all the dosimetric criteria, providing flexibility for further optimization and accommodating the PSVs. However, in more complex planning scenarios where it is difficult to meet important dosimetric criteria, the magnitude of the PSVs may have significant impacts on adaptive planning. Therefore, caution should be exercised when applying the conclusions of this study to more complex clinical scenarios, and efforts should always be made to ensure optimal immobilization to minimize the PSVs.

## 5 CONCLUSION

We analyzed the PSVs for brain, pancreas, and pelvis patients treated on the Elekta Unity system. The lack of customizable body molds, the absence of lateral lasers, the use of BBs and skin tattoos for marking locations, and the internal organ motion (including variation in bladder/rectum filling, etc.) all contributed to the larger variations observed on Elekta Unity compared with conventional linacs. Since the treatment couch cannot move to correct setup errors, the system adapts the treatment plan based on a localization MR scan. Using five prostate and five pancreas plans, we studied the impact of the magnitude of the PSVs on the performance of both the ATP and ATS workflows. PSVs of up to 3 cm in the LR and SI directions were simulated by shifting the reference MR scan. The ATS workflow took significantly longer than the ATP workflow. There was a slight increase in plan MUs as the setup variations increased in the ATP workflow for the prostate plans (10% increase with 3 cm shift in the LR and SI directions). No other trend related to the magnitude of the PSVs was observed. All adaptive plans met target coverage and the OAR constraints. A few ATP plans and ATS plans produced plans with hotspots slightly exceeding the tolerance. No advantages were identified if the treatment couch were able to move in the SI direction. The adaptive workflows can effectively account for the PSVs in the studied cases. However, effort should still be made to ensure optimal immobilization to mitigate rotation errors and intra‐fractional motion.

## AUTHOR CONTRIBUTIONS

Author contributions—all authors contributed in the design, data collection, manuscript preparation, and its review and editing. Maggie Yan and Erika Kollitz performed data analysis and plan simulation. Kathryn Hitchcock and Alexandra De Leo provided clinical supervision and input to the project. Sheng‐Hsuan Sun, Amanda Schwarz, Luke Maloney, Jonathan Li, Chihray Liu, Guanghua Yan recorded the data during patient treatment and provided study design.

## CONFLICT OF INTEREST STATEMENT

The authors declare no conflicts of interest.

## References

[1] Benedict SH , Yenice KM , Followill D , et al. Stereotactic body radiation therapy: the report of AAPM Task Group 101. Med Phys. 2010;37(8):4078‐4101. 20879569 10.1118/1.3438081

[2] Tsai P , Liu C , Kahler DL , Li JG , Lu B , Yan G . A self‐checking treatment couch coordinate calculation system in radiotherapy. J Appl Clin Med Phys. 2020;21(1):43‐52. 10.1002/acm2.12771 PMC6964758 31737999

[3] Cuccia F , Alongi F , Belka C , et al. Patient positioning and immobilization procedures for hybrid MR‐Linac systems. Radiat Oncol. 2021;16(1):183. 34544481 10.1186/s13014-021-01910-6 PMC8454038

[4] Rostami A , Robatjazi M , Javadinia SA , Shomoossi N , Shahraini R . The influence of patient positioning and immobilization equipment on MR image quality and image registration in radiation therapy. J Appl Clin Med Phys. 2024;25(2):e14162. 37716368 10.1002/acm2.14162 PMC10860429

[5] Konnerth D , Eze C , Nierer L , et al. Novel modified patient immobilisation device with an integrated coil support system for MR‐guided online adaptive radiotherapy in the management of brain and head‐and‐neck tumours. Tech Innov Patient Support Radiat Oncol. 2021;20:35‐40. 34841095 10.1016/j.tipsro.2021.11.002 PMC8605429

[6] Winkel D , Bol GH , Kroon PS , et al. Adaptive radiotherapy: the Elekta Unity MR‐linac concept. Clin Transl Radiat Oncol. 2019;18:54‐59. 31341976 10.1016/j.ctro.2019.04.001 PMC6630157

[7] Werensteijn‐Honingh AM , Kroon PS , Winkel D , et al. Feasibility of stereotactic radiotherapy using a 1.5 T MR‐linac: multi‐fraction treatment of pelvic lymph node oligometastases. Radiother Oncol. 2019;134:50‐54. 31005224 10.1016/j.radonc.2019.01.024

[8] Dunkerley DAP , Hyer DE , Snyder JE , et al. Clinical implementational and site‐specific workflows for a 1.5T MR‐Linac. J Clin Med. 2022;11(6):1662. 35329988 10.3390/jcm11061662 PMC8954784

[9] Raaymakers BW , Lagendijk JJ , Overweg J , et al. Integrating a 1.5 T MRI scanner with a 6 MV accelerator: proof of concept. Phys Med Biol. 2009;54(12):N229‐N237. 19451689 10.1088/0031-9155/54/12/N01

[10] Yang J , Vedam S , Lee B , et al. Online adaptive planning for prostate stereotactic body radiotherapy using a 1.5 Tesla magnetic resonance imaging‐guided linear accelerator. Phys Imaging Radiat Oncol. 2021;17:20‐24. 33898773 10.1016/j.phro.2020.12.001 PMC8057955

